# THE INTERNET IS A BOY’S CLUB?: ANALYZING GENDER DIFFERENCES IN COMPUTER & INTERNET ADOPTION BY OLDER ADULTS

**DOI:** 10.1093/geroni/igz038.3167

**Published:** 2019-11-08

**Authors:** Derek A Wilson

**Affiliations:** University of Kansas, Lawrence, Kansas, United States

## Abstract

The realization of technology’s exponential advancement has been noted in recent years. With ever advancing technologies becoming more integrated in our everyday lives, we must adapt and learn to utilize these new technologies in order to maintain a presence in society. One group that has been stereotyped as struggling to adopt and learn the processes involved with advancing technologies is that of the older adult population. However, previous literature tends to suggest that there are also gendered differences in the adoption of different forms of modern and advancing technologies among older adults. While some articles state that older adult women have been reported to use social media than their male counterparts, conflicting previous literature states that older adult women use newer technologies less, but are more creative in their uses. With these conflicting reports, there comes a need for proper analyses on the gendered use of modern technology among older adults. Using data from the 2015 Current Population Survey (CPS) Computer and Internet Use Supplement, different demographic factors are analyzed for influences on use of technologies. The older adult age cohorts analyzed include the Baby Boomer and the Silent Generation age cohorts. This analysis includes of controlling for additional factors such as regional residence, educational attainment, and other social location variables. Contrary to much of the existing literature, there is little difference in the adoption of computer and internet technologies by older adults. Additionally, the analyses are performed on the Generation X and Millennial age cohorts for comparison.

